# Umbilical Cord Blood Transplantation: Challenges and Future Directions

**DOI:** 10.1002/sctm.17-0069

**Published:** 2017-04-29

**Authors:** Karen Ballen

## Abstract

Since the first successful allogeneic transplants performed in Seattle 50 years ago, the field of transplantation has evolved considerably, with improvements in human leukocyte antigen typing, patient selection, reduced intensity regimens, and graft‐versus‐host disease prophylaxis. A major breakthrough has been the availability of more donor options, first via the National Marrow Donor Program—Be the Match [Biol Blood Marrow Transplant 2008;14:2–7]. Then, in the 1990s, unrelated umbilical cord blood transplantation became available, first for children and then for adults [New Engl J Med 1996;35:157–166]. More recently mismatched unrelated transplants and haploidentical donor options became available [Blood 2011;118:282–288]. In 2017, there is a donor for almost every patient who needs a transplant. In this review, we will discuss the state of the science (and art) of cord blood transplant, focusing on successes, challenges, and future directions. Stem Cells Translational Medicine *2017;6:1312–1315*

**Significance Statement**

From the first UCBT in 1988, the field of UCBT has evolved considerably. UCBT is now a successful treatment option for both pediatric and adult patients with a variety of hematologic diseases, and transplant outcomes continue to improve with better HLA matching, UCB unit selection, refinement of conditioning regimens, and expanded supportive and infection prevention regimens.

## Umbilical Cord Blood Banking

An estimated 700,000 umbilical cord blood (UCB) units have been donated for public use. Given the association between cell dose and engraftment, many centers are choosing larger units (based on total nucleated cell count or CD34+ dose) for transplantation, even for pediatric patients 1, 2. There are currently more than 100 UCB banks collecting units for public use in North America, South America, Australia, Europe, Asia, and the Middle East. In the United States, federal regulations require that a UCB must either be licensed by the Food and Drug Administration (FDA) or used under an Investigational New Drug (IND) protocol.

Private or family UCB banks collect units for family use; an estimated 4 million units have been stored for private use 3. In Europe, hybrid UCB banking is an innovative strategy to use private donations to fund the public banking side, and families can opt to have privately stored UCB units available for patients in need 4.

### Major Challenges in Umbilical Cord Blood Banking

1. Regulatory issues, such as licensure, have increased the cost to bank UCB units.
2. Less than 1 in 10 stored UCB units are used for transplantation, also increasing the costs 5.
3. Some obstetrical practices, such as delayed cord clamping, may affect the volume and cell dose collected 6.

### Future Directions in Umbilical Cord Blood Banking

Umbilical cord blood banks have adapted to economic challenges by carefully selecting units to human leukocyte antigen (HLA) type, freeze, and store. Many banks have increased their minimum cell dose to 125 or 150 × 10^7^ nucleated cells before processing UCB units 5. In addition, the use of automated freezing practices is more widespread 7. Innovative ways to use public and private funds to support UCB initiatives are under way. Newer ways to thaw UCB cells at the transplant center, using a dilution and no‐wash method, may increase cell recovery 8, 9.

## Cord Blood Transplant for Hematologic Diseases

Umbilical cord blood transplant (UCBT) is potentially curative therapy for patients with leukemia, lymphoma, myeloma, myeloproliferative disorders, genetic diseases, and disorders of metabolism. UCB is particularly important for patients of non‐Western European ancestry, because these patients have a difficult time finding a matched volunteer donor in the donor registry 10. The use of double cord blood transplant and reduced intensity regimens in adults has led to increased use in older patients and reduced transplant‐related mortality 11, 12.

### Major Challenges in Cord Blood Transplant for Hematologic Diseases

1. Engraftment and immune reconstitution are delayed, which leads to an increased risk of infection 13.
2. The cost of acquisition of two cord blood grafts (for double cord blood transplant in adults) can be $80,000, in addition to the cost of the transplant admission and immediate post‐transplant care 14.
3. Relapse of the primary disease remains the major cause of death for patients post‐transplant.

### Future Trends in Cord Blood Transplant for Hematologic Diseases

There are many techniques under investigation to improve immune reconstitution and engraftment (Table 1). Expansion trials include efforts with mesenchymal progenitor cell expansion, which showed a neutrophil engraftment of 15 days, improved from a historical control of 24 days 15. Using the notch ligand Delta 1, Delaney and colleagues improved neutrophil engraftment to 16 days 16. This work has now been extended to use an “off‐the‐shelf” non‐HLA‐matched expanded UCB product, and a phase II study is under way (NCT01690520). The use of copper chelation led to the development of the NiCord product, which showed a one‐year overall survival of 82% and 11 days to neutrophil engraftment in a phase I study 17. The product recently obtained breakthrough designation from the FDA, and a phase III registration trial comparing expanded versus unexpanded UCB is in progress (NCT02730299). While expansion studies are promising, the studies have been limited by small sample size and complex technology that may be difficult to export to other centers.

**Table 1:** Strategies to improve engraftment and immune recovery

Agent	Mechanism	Author	*n*	Days to ANC > 500	Current trial
Nicotinamide	Inhibit enzymes that require NAD+	Horwitz et al. [17]	11	13	NCT02730299
Notch	Inhibit differentiation	Delaney et al. [16]	10	16	NCT01690520
Mesenchymal stem cells	Improve stroma	De Lima et al. [15]	31	15	NCT01854567
Prostaglandin E2	Homing	Cutler et al. [11]	12	17	
FT‐VI	Fucosylation	Popat et al. [18]	7	14	NCT01471067
Sitagliptin	DPP‐IV inhibition	Farag et al. 45	24	21	NCT01720264
Intrabone marrow	Homing	Kurita et al. [21]	15	17	

Abbreviations: ANC, absolute neutrophil count; FT, fucosyltransferase; DPP, dipeptidyl peptidase; NAD+, nicotinamide adenine dinucleotide.

Another approach is to improve homing of the infused UCB cells to the bone marrow. The Boston group has used prostaglandin E2 to upregulate CXCR4 expression and has shown improved engraftment 11. Other strategies include the use of fucosylation, hyperbaric oxygen, and direct intramarrow injection of the UCB cells 18, 19, 20, 21. Additional efforts to reduce infection include the use of cytotoxic T lymphocytes to decrease viral infection 22.

If expansion and homing techniques prove successful, this work may decrease the cost of cord blood transplantation by eliminating the need for the second UCB unit. Single unit UCBT has been shown to be equivalent to double UCBT in children and may also be acceptable in younger adults 4, 23.

Although relapse remains the major cause of death, recent work from the Seattle group has shown that for patients with minimal residual disease, UCBT is associated with a lower risk of relapse than for patients receiving unrelated donor transplants 24. In addition, for transplant in general, targeted post‐transplant maintenance therapy, such as for patients with FLT3‐positive acute myeloid leukemia, may decrease the risk of relapse 25, 26.

## Cord Blood Transplantation in Regenerative Medicine

An exciting new development is the use of either autologous or unrelated UCBT for nontraditional applications (outside of oncology) in neurology, endocrinology, and cardiology, for diseases that have significant worldwide impact. Compared with stem cells obtained from adult bone marrow harvests, UCB stem cells have greater proliferative potential and longer telomeres 27. UCB has been used to treat neurologic conditions, including cerebral palsy, hypoxic ischemic encephalopathy, traumatic brain injury, and autism 28. In cardiovascular disease, UCB‐derived mesenchymal stem cells are in clinical trials for dilated cardiomyopathy and ischemic disease 29.

### Major Challenges in Cord Blood Transplantation in Regenerative Medicine

1. Trial endpoints may be more difficult to quantitate than in hematologic malignancies, for example improved function in cerebral palsy 30.
2. There are significant regulatory hurdles for large‐scale use of these products.
3. Use of autologous UCB units for regenerative medicine indications may affect UCB public banking.

### Future Trends in Cord Blood Transplantation in Regenerative Medicine

This is a fast‐moving field with several clinical trials under way. In cerebral palsy, intravenous autologous UCB infusions have been administered safely 30. Neurodevelopmental improvement has been seen in a study of 57 patients treated with G‐CSF with or without autologous peripheral blood stem cells 31. Allogeneic infusions have also been used; 47 patients with severe cerebral palsy were treated safely with unmatched allogeneic UCB cells, given both intravenously and intrathecally 32. Gross motor function scores improved, and there was no graft‐versus‐host disease 33.

Approximately 15 million babies are born preterm worldwide, and these babies are at much higher risk of neurodevelopment abnormalities, likely related to hypoxia‐ischemia 34. Rat models have shown an improvement in motor function after transplantation of human cord blood 35. Clinical trials using UCB are ongoing at Duke and National University Hospital, Singapore (NCT00593242) 28, 36.

In cardiovascular disease, UCB mesenchymal stem cells secrete cytokines that stimulate angiogenesis 37. In rat models of myocardial infarction, UCB‐derived mesenchymal stem cells have been shown to decrease infarct size, improve cardiac function, and promote angiogenesis via activating platelet‐derived growth factor D 38, 39.

Human UCB‐derived cells are also being studied to treat inflammatory bowel disease, corneal disease, renal disease, and collagen‐induced arthritis 40, 41, 42. A clinical trial in 45 patients with hepatitis B‐induced liver disease has shown a benefit to UCB‐derived mesenchymal stem cells 43. A partial listing of available clinical trials for UCB‐derived cells for regenerative medicine applications is shown in Table 2.

**Table 2:** Selected active and recruiting regenerative medicine human cord blood trials

Disease	Agent	Investigator	Ages (years)	Current trial
Autism	Auto or Allo UCB	Kurtzberg	2–7	NCT02847182
Cerebral palsy	Auto UCB	Carroll	1–12	NCT01072370
Cerebral palsy	Auto UCB and G‐CSF	Lee	2–10	NCT02866331
Ischemic stroke	Allo UCB	Kurtzberg	18–90	NCT03004976
Chronic ischemic cardiomyopathy	UCB‐derived mesenchymal stem cells	Dai	35–65	NCT02635464
Crohn's disease	UCB‐derived stem cells	Lee	20–70	NCT02000362

Abbreviations: G‐CSF, granulocyte colony‐stimulating factor; UCB, umbilical cord blood;

## Conclusion

From the first UCBT in 1988, the field of UCBT has evolved considerably 44. UCBT is now a successful treatment option for both pediatric and adult patients with a variety of hematologic diseases, and transplant outcomes continue to improve with better HLA matching, UCB unit selection, refinement of conditioning regimens, and expanded supportive and infection prevention regimens. Exciting new applications in the field of cardiology, neurology, autoimmune disease, and ophthalmology should make major health advances in the next 10 years.
